# Non-surgical pneumoperitoneum after oro-genital intercourse^☆^

**DOI:** 10.1016/j.ijscr.2013.08.022

**Published:** 2013-09-25

**Authors:** Shamir O. Cawich, Peter B. Johnson, Eric Williams, Vijay Naraynsingh

**Affiliations:** a Department of Clinical Surgical Sciences, University of the West Indies, St Augustine Campus, Trinidad and Tobago; b Department of Surgery, University of the West Indies, Mona Campus, Jamaica

**Keywords:** Pneumoperitoneum, Peritonitis, Non-surgical, Emergency, Benign

## Abstract

**INTRODUCTION:**

In many cases, a pneumoperitoneum is due to air escaping from a perforated hollow viscus or surgical intervention but there are increasing reports of non-surgical causes.

**PRESENTATION OF CASE:**

We report a case where a pneumoperitoneum was identified after oro-genital sexual intercourse.

**DISCUSSION:**

There were nineteen reported cases of non-surgical pneumoperitoneum from gynaecologic causes up to May 2013. We report an additional case four hours after oro-genital intercourse. Close clinical observation and symptomatic treatment are usually all that is required but operative interventions should be considered if the patient develops abdominal pain, peritoneal signs, fever or leukocytosis during observation.

**CONCLUSION:**

This adds to the world literature on non-surgical pneumoperitoneum from oro-genital intercourse. Clinicians should be aware of this condition and focus on medical-sexual history as this information could prevent a patient from being exposed to expensive diagnostics and invasive operative treatments. Patients should also be educated about the mechanisms to avoid future possible diagnostic dilemmas.

## 1 Introduction

In many cases a pneumoperitoneum is due to air escaping from a perforated hollow viscus or surgical intervention. This is considered a surgical emergency, demanding prompt control of the perforation and peritoneal toilet. However, there are increasing reports of non-surgical pneumoperitoneum, a condition in which radiographs demonstrate free peritoneal air. These may lead to unnecessary laparotomy but, if correctly diagnosed, can be managed successfully by observation alone.^1^ We report one such case where a pneumoperitoneum was identified after oro-genital sexual intercourse.

## 2 Presentation of case

A 21-year-old woman with a body mass index of 22.3 presented to hospital complaining of sudden onset right-sided abdominal pain. She was dehydrated and pyrexic at 100°F. The abdomen was asymmetrically distended and tender with peritonitis on the right side. Leukocytosis was present with a white cell count at 16.1 × 10^3^ units. Serum electrolytes, urea, creatinine and amylase levels were normal.

**Fig. 1:**
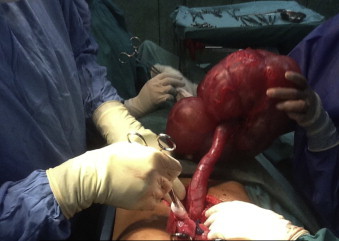
Right hydronephrosis and hydroureter encountered at laparotomy.

She was taken to the operating room for abdominal exploration through a midline laparotomy incision. An enlarged right polycystic kidney was encountered with bossellated surface (Fig. 1). There was pyonephrosis with a thin, translucent renal cortex and a grossly dilated ureter present down to the bladder. The left kidney was mildly enlarged with a normal ureter. No further abnormalities were detected at any other intra-abdominal viscera.

**Fig. 2:**
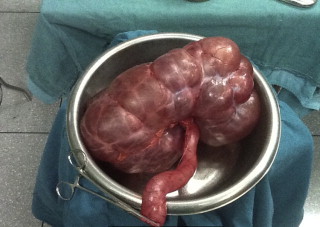
Nephrectomy specimen.

Since there was no discernible renal parenchyma and an obvious pyonephrosis, a right nephroureterectomy was performed (Fig. 2). She was discharged home 5 days post-operatively after an uneventful recovery period.

**Fig. 3:**
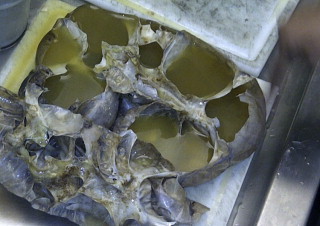
Gross examination of right kidney reveals cystic transformation and cortical replacement.

Pathologic examination revealed a 12 cm × 20 cm × 32 cm right kidney that weighed 4870 g. Histology confirmed pyonephrosis of the kidney with no normal renal cortex. A thin translucent membrane <1 mm mural thickness represented the renal capsule (Fig. 3). There were multiple cysts lined by flattened cuboidal cells and marked inflammatory cell infiltrates. Circumferential scarring at the distal ureter near the vesico-ureteric junction was responsible for the hydro-ureter. Urinary cultures revealed significant growth of *Escherichia coli* Spp but no gas forming organisms were present.

**Fig. 4:**
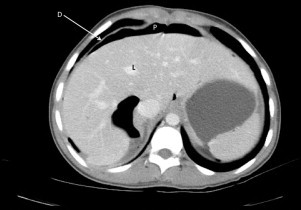
Axial CT slice of upper abdomen demonstrating a pneumopertioneum (P) that is contained by the diaphragm (D) and liver (L).

**Fig. 5:**
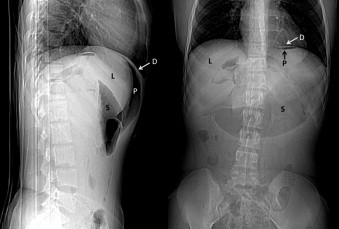
CT scout films of the abdomen demonstrating a pneumopertioneum (P) that is contained by the diaphregram (D) and the liver (L). A dilated stomach (S) is also seen.

Eight weeks post-operatively, she was sent for elective CT urogram to evaluate the left kidney function. To our surprise, a large pneumoperitoneum was present, predominantly on the right side (Figs. 4 and 5).

**Fig. 6:**
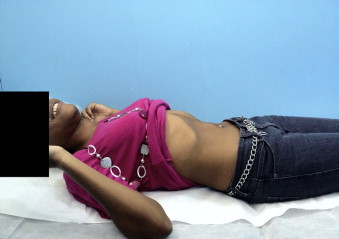
Index patient immediately post CT scanning demonstrating a flat abdomen.

Clinically she was well. There was no history of colonoscopy, cystoscopy or any other invasive interventions since the time of operation. The abdomen was soft, flat and non-tender (Fig. 6). She had a white cell count of 4.3 × 10^6^ dl^–1^ and no evidence of metabolic acidosis. Upon further detailed questioning she admitted to engaging in sexual activity approximately four hours prior to CT scanning. Specifically, there was cunnilingus lasting approximately 15 min and that was followed by regular vaginal intercourse. She denied deliberate vaginal insufflation, anal intercourse or any other sexual acts.

She was admitted for clinical observation. No antibiotics were prescribed. Over the subsequent 48 h, she remained clinically well with no fever, abdominal signs or leukocytosis. She was discharged and remained well up to six months later. As she remained clinically well, a conscious decision was made not to subject this patient to any form of repeat imaging to reassess the pneumoperitoneum.

## 3 Discussion

A pneumoperitoneum can be detected on plain radiographs in 60% of patients after open surgery and 25% after laparoscopy^1–2^ but we expect a progressive reduction in volume as gas is resorbed across the peritoneum.^2–4^ On follow-up radiographs, there is usually complete reabsorption of room air within 5 days of open surgery.^5^ Carbon dioxide is much more rapidly absorbed at average rates of 37 ml/min, with complete resolution of pneumoperitoneum within 2–4 h of laparoscopy.^4^

Computed tomography scans are far more sensitive for a pneumoperitoneum than plain radiographs.^2,6^ A pneumoperitoneum can be detected on CT in >85% of post-operative patients at day 3 and >50% at day 6.^7^ Additionally, lean adults tend to have prolonged duration of pneumoperitoneum after abdominal procedures than over-weight adults.^8^ Although our patient had two recognized predispositions (BMI of 22.3 and recent open surgery), we considered it unlikely that this was secondary to her laparotomy because there has never been a post-operative pneumoperitoneum reported 8 weeks after laparotomy.

Excluding post-operative cases, the presence of a pneumoperitoneum signals the presence of a perforated hollow viscus in 90%^3^ to 95%^2^ of cases. These patients require emergent surgical treatment that is directed at controlling the perforation and achieving peritoneal toilet. In a minority of cases, a pneumoperitoneum is detected in the absence of clinical signs that suggest an intra-abdominal emergency. The terms benign,^9^ spontaneous^10–13^ and non-surgical^13–15^ pneumoperitoneum have been applied to these cases.

Daly^3^ classified non-surgical pneumoperitoneum into abdominal, thoracic and pelvic causes. Compared to the other causes of non-surgical pneumoperitoneum, a pelvic (gynaecologic) cause is uncommon. Mularski et al.^2^ performed a systematic review of world literature on non-surgical pneumoperitoneum and identified only 15 reported cases that were due to gynecologic causes up to the year 2000. We performed a Pubmed search using the keywords “spontaneous”, “non-surgical”, “benign”, “pelvic” and “gynaecologic” in May 2013 and encountered 4 additional cases of non-surgical pneumoperitoneum from gynaecologic causes.^16–19^

The common gynaecologic causes include pelvic examinations^2–4,18,20^, post-partum knee-chest exercises,^3,21,22^ coitus,^3,4,16,19,23,24,25^ oro-genital sex,^26–29^ vaginal douching,^3,4,30^ pelvic inflammatory disease,^31^ hysterosalpingography^31^ and water skiing.^32^ The essential mechanism is the passage of air through the vagina, cervix, uterus and fallopian tubes into the peritoneum^3,4^ or through a vaginal stump opening in patients who have had hysterectomies.^25,33,34^

The only identifiable cause in our patient was the recent history of oro-genital intercourse 4 h prior to CT. This reinforces the need to take a thorough history because most patients will not volunteer sexual histories since they cannot readily make a link between sexual practices and their symptoms or radiographic findings. This may prevent the exposure of these patients to expensive investigations and non-therapeutic laparotomies.^3,4^

This should still be considered a diagnosis of exclusion since peritonitis may be masked in immunocompromised patients.^3^ Once the diagnosis is established, however, close clinical observation and symptomatic treatment are usually all that is required.^4,15^ Antibiotics are not indicated as there is no infective pathophysiologic mechanism.^4^ Operative intervention should be considered if the patient develops abdominal pain, peritoneal signs, fever or leukocytosis during observation.

## 4 Conclusion

This case adds to the world literature on non-surgical pneumoperitoneum from oro-genital intercourse. Clinicians should be aware of this condition and focus on medical-sexual history as this information could prevent a patient from being exposed to expensive diagnostics and invasive operative treatments. Patients should also be educated about the mechanisms to avoid future possible diagnostic dilemmas.

## Conflict of interest statement

There are no potential sources of conflict declared by any of the authors.

## Funding

None.

## Ethical approval

Written informed consent was obtained from the patient for publication of this case report and case series and accompanying images. A copy of the written consent is available for review by the Editor-in-Chief of this journal on request.

## Author contributions

SOC conceptualized this manuscript, collected data and wrote the manuscript.

PBJ assisted in writing this manuscript and checked it for intellectual content.

EWW assisted with data collection and analysis of the manuscript.

VN assisted with data collectin, writing and analysis of the intellectual content in this paper.

## References

[1] Mularski R.A., Ciccolo M.L., Rappaport W.D. (1999). Nonsurgical causes of pneumoperitoneum. West J Med.

[2] Mularski R.A., Sippel J.M., Osborne M.L. (2000). Pneumoperitoneum: a review of non-surgical causes. Crit Care Med.

[3] Daly B.D., Guthrie J.A., Couse N.F. (1991). Pneumoperitoneum without peritonitis. Postgrad Med J.

[4] Jacobs V.R., Mundhenke C., Maass N., Hilpert F., Jonat W. (2000). Sexual activity as a cause for non-surgical pneumoperitoneum. JSLS.

[5] Nielsen K.T., Lund L., Larsen L.P. (1997). Duration of postoperative pneumoperitoneum. Eur J Surg.

[6] Stapakis J.C., Thickman D. (1992). Diagnosis of pneumoperitoneum: abdominal CT vs. upright chest ﬁlm. J Comput Assist Tomogr.

[7] Earls J.P., Dachman A.H., Colon E. (1993). Prevalence and duration of postoperative pneumoperitoneum: sensitivity of CT vs left lateral decubitus radiography. AJR Am J Roentgenol.

[8] Bryant L.R., Wolf J.F., Kloecker R.J. (1963). A study of the factors affecting the incidence and duration of post-operative pneumoperitoneum. Surg Gynecol Obstet.

[9] Ecker M.D., Goldstein M., Hoexter B. (1977). Benign pneumoperitoneum after ﬁberoptic colonoscopy: a prospective study of 100 patients. Gastroenterology.

[10] Madura M.I., Craig R.M., Shields T.W. (1982). Unusual causes of spontaneous pneumoperitoneum. Surg Gynecol Obstet.

[11] Matsuda M., Nishikawa N., Okano T., Hoshi K., Suzuki A., Ikeda S. (2003). Spontaneous pneumoperitoneum: an unusual complication of systemic reactive AA amyloidosis secondary to rheumatoid arthritis. Amyloid.

[12] Derveaux K., Penninck F. (2003). Recurrent spontaneous pneumoperitoneum: a diagnostic and therapeutic dilemma. Acta Chir Belg.

[13] Marwah S., Gupta R., Dhall J.C. (2002). Non-surgical spontaneous pneumoperitoneum: a case report. Indian Practitioner.

[14] Rowe N.M., Kahn F.B., Acinapura A.J., Cunningham J.N. (1998). Nonsurgical pneumoperitoneum: a case report and a review. Am Surg.

[15] Hoover E.L., Cole G.D., Mitchell L.S., Adams C.Z., Hassett J. (1992). Avoiding laparotomy in nonsurgical pneumoperitoneum. Am J Surg.

[16] Im D.D., Pak P.S., Cua B., Feinberg E. (2012). Pneumoperitoneum after sexual assault in a patient who had hysterectomy 30 years ago: case report. J Emerg Med.

[17] Eskandar O., El Badawy S., Bennett S. (2007 Apr). Spontaneous/non-surgical pneumoperitoneum in a 34-week-pregnant patient. Aust N Z J Obstet Gynaecol.

[18] Vlachou P.A., Aslam M., Ntatsios A., Anagnostopoulos G.K., Murphy P. (2007). Non-surgical pneumoperitoneum following cervical smear test. Eur J Obstet Gynecol Reprod Biol.

[19] Jacobs V.R., Mundhenke C., Maass N., Hilpert F., Jonat W. (2000). Sexual activity as cause for non-surgical pneumoperitoneum. JSLS.

[20] Cass L.J., Dow E.C., Brooks J.R. (1966). Pneumoperitoneum following pelvic examination. Am J Gastroenterol.

[21] Dodek S.M., Friedman J.M. (1953). Spontaneous pneumoperitoneum. Obstet Gynecol.

[22] Lozman H., Newman A.J. (1956). Spontoneous pneumoperitoneum occurring during post partum exercises in the knee–chest position. Am J Obstet Gynecol.

[23] Christiansen W.C., Danzl D.F., McGee H.J. (1980). Pneumoperitoneum following vaginal insufﬂation and coitus. Ann Emerg Med.

[24] Spaulding L.B., Gallop D.A. (1979). Pneumoperitoneum after hysterectomy. JAMA.

[25] Tabrinsky J., Mallin L.P., Smith J.A. (1972). Pneumoperitoneum after coitus: a complication due to uterine prolapse after vaginal hysterectomy. Obstet Gynecol.

[26] Collins K.A., Davis G.J., Lantz P.E. (1994). An unusual case of maternal-fetal death due to vaginal insufflation of cocaine. Am J Forens Med Path.

[27] Fyke F.E., Kazmier F.J., Harms R.W. (1985). Venous air embolism: life threatening complication of orogenital sex during pregnancy. Am J Med.

[28] Freeman R.K. (1970). Pneumoperitoneum from oral-genital insufﬂation. Obstet Gynecol.

[29] Varon J., Laufer M.D., Sternbach G.L. (1991). Recurrent pneumoperitoneum following vaginal insufﬂation. Am J Emerg Med.

[30] Walker M.A. (1942). Pneumoperitoneum following a douche. J Kans Med Soc.

[31] Miller R.E. (1973). The radiological evaluation of intraperitoneal gas (pneumoperitoneum). Crit Rev Clin Radiol Nucl Med.

[32] Johnson L.B. (1993). A case report: peritonitis after water skiing. Lakartidningen.

[33] Borowski G.D., Veloso A., Nothmann B.J. (1983). Recurrent pneumoperitoneum after hysterectomy. J Clin Gastroenterol.

[34] Nicolsen S.C., Gillmer M.D.G. (1993). Management of postcoital pneumoperitoneum following hysterectomy. J Obstet Gynecol.

